# Chemically Modified Clay Adsorbents Used in the Retention of Protein and Polyphenolic Compounds from Sauvignon Blanc White Wine

**DOI:** 10.3390/nano14070588

**Published:** 2024-03-27

**Authors:** Andreea Hortolomeu, Diana Carmen Mirila, Ana-Maria Roșu, Florin Marian Nedeff, Iuri Scutaru, Dorel Ureche, Rodica Sturza, Adriana-Luminița Fînaru, Ileana Denisa Nistor

**Affiliations:** 1 Department of Chemical and Food Engineering, Faculty of Engineering, “Vasile Alecsandri” University of Bacau, 157, Calea Marasesti, 600115 Bacau, Romaniamiriladiana@ub.ro (D.C.M.); ana.rosu@ub.ro (A.-M.R.); adrianaf@ub.ro (A.-L.F.); 2 Department of Environmental Engineering and Mechanical Engineering, Faculty of Engineering, “Vasile Alecsandri” University of Bacau, 157, Calea Marasesti, 600115 Bacau, Romania; florin_nedeff@ub.ro; 3 Department of Oenology and Chemistry, Faculty of Food Technology, Technical University of Moldova, 9/9 Studentilor Street, MD-2045 Chisinau, Moldova; 4 Department of Biology, Ecology and Environmental Protection, Faculty of Sciences, “Vasile Alecsandri” University of Bacau, 157, Calea Marasesti, 600115 Bacau, Romania; dureche@ub.ro

**Keywords:** bentonite, wine, proteins, polyphenols, tannins

## Abstract

During the manufacturing process of white wine, various physicochemical reactions can occur and can affect the quality of the finished product. For this reason, it is necessary to apply different treatments to minimize distinct factors such as protein instability and pinking phenomenon, which can affect the organoleptic properties of wines and their structure. In this work, a new method for the preparation of a sorbent-type material is presented through the fractional purification of native bentonite in three fractions (Na-BtF1, Na-BtF2, and Na-BtF3). Furthermore, the influence of the prepared sorbents on pH, conductivity, and amino nitrogen level was analyzed. The absorbents prepared and tested in wine solutions were characterized using the following physico-chemical methods: Brunauer–Emmett–Teller and Barrett–Joyner–Halenda (BET-BJH) method, X-ray diffraction (XRD) technique, and transform-coupled infrared spectroscopy Fourier with attenuated total reflection (FTIR-ATR). Following the analyses carried out on the retention of protein content and polyphenolic compounds, it was found that materials based on natural clay have suitable adsorption properties.

## 1. Introduction

The presence of tannins mixed with proteins in white wine can form a negative hydrophobic colloid that can flocculate in the presence of cations. The aggregation of the proteins present in white wine is usually associated with a change in the protein structure due to the elimination of water. Due to the possibility of the phenomenon of flocculation in the glass of white wines kept at an elevated temperature above 25 °C, degradation of the finished product occurs through the appearance of the protein aggregation process.

Most of the proteins responsible for the instability of white wines come from the raw material [1]. The haze phenomenon often takes place during the wine storage process. This phenomenon occurs because of the deformation of the proteins present in the raw material and in the final product (wine). The molecular weights of the proteins are low, up to 40 kDa [2], and are resistant to the pH of the wine and to the proteolysis process [3]. However, the molecular structure of wine, consisting of polyphenols, polysaccharides, and ethanol, usually produces a complex interaction and leads to the apparition of haze phenomenon in white wine [4,5,6].

The content of unstable proteins depends on many factors, such as grape variety (Sauvignon Blanc white wine variety is rich in thermo-unstable proteins), maturity, viticultural practices, pre-fermentation techniques, etc. [7,8,9]. In the oenology laboratories, various tests are carried out to assess the risk of protein disturbance under different conditions: at high temperatures or in the presence of different compounds (trichloroacetic and phosphomolybdic acids, ammonium sulfate, etc.) [10]. Bentonite is a mineral resulting from the decomposition of some volcanic rocks. It is a hydrated aluminum silicate with a phyllite structure like montmorillonite, with the property of swelling in a dispersed environment. Immersed in wine, bentonite forms a colloidal dispersion with negatively charged particles, which have the role of fixing the proteins in the wine at an optimal environmental pH (pH 3.0–3.3) [11,12]. Due to the high density of bentonite, it will settle to the bottom of the storage vessel, being easily recovered from the wine [13].

In practice, the use of sorbents of this type in the process of obtaining wines can affect their quality, such as reducing the aromatic potential of white wine [14]. Instead, the use of bentonite treatment of young white wines has a favorable effect from an organoleptic point of view, highlighting the properties of the finished product on the taste and smell of the wine. Bentonite also contributes to microbiological stabilization in winemaking by removing unwanted microorganisms. Another factor that affects the quality of white wines is the browning phenomenon [15]. The browning phenomenon of wines often occurs during the technological process due to non-compliance with the working conditions during the pre-fermentation process of the must [10].

According to Revi M. and Serra-Cayuela, A. [16,17], polyphenolic compounds manage the formation of oxidation reactions. Through the research carried out, it was determined that the main polyphenolic compounds of white wine that are prone to these reactions are flavonoids (yellow or colorless substances) and catechins (3-flavanols, main substances of tannins in grape seeds) [18,19]. A current technique for stabilizing white wines against oxidation of polyphenolic substances is hyper-oxygenation. The procedure presented by Romanini E. and Lingua, M.S. [20,21] is based on the controlled oxidation of polyphenols and must be performed immediately after the pressing and deburring stages.

The aim of this paper is to present the effects of chemically modified bentonite on the adsorption of protein and polyphenolic compounds from white wines. Due to its availability in nature and its suitable adsorption capacity in wine, native bentonite was chosen as the base material.

The treatment and purification of montmorillonite have been researched for years. Different methods of enrichment, activation, and application in wine have already been tested [22,23]. Different Na-activated bentonite labels have also been studied in wine fining [24]. Table 1 presents some of the used materials known as stabilizing agents.

The novelty of this article is the use of autochthonous bentonite collected fractionally and used in winemaking as improved materials that keep white wine bio-polymers prone to flocculation and premature oxidation. The aim of this work was to study the retention degree of proteins and polyphenolic compounds in clay materials. In this work, thermal stability tests, oxidizability tests, and spectrophotometric and potentiometric analyses were applied.

## 2. Materials and Methods

### 2.1. Materials

The white wine tested in this work is from the European Sauvignon Blanc grape variety, obtained in the Microvinification Section of Oenology at the Technical University of Moldova in the Republic of Moldova. Before being tested, the wine was filtered using a 0.45 µm microfilter. An Analytic Jena Specord 250 Plus UV-VIS device (Berlin, Germany) was used for spectrophotometric determinations. Three percent hydrogen peroxide (H_2_O_2_) solution, 0.2 N sodium hydroxide solution, and formalin mixture were used in the experiments in this paper. Four different bentonite solutions were used as absorbent material: bentonite (BtB) obtained from Valea Chioarului (Maramureș, Romania) and the three fractions named below: Na-BF1 the first fraction, Na-BF2 the second fraction, and Na-BF3 fraction three. Double-distilled water and sodium chloride were used to perform the bentonite purification process. To find the physicochemical parameters of the wine samples, a WTW Inolab 7110 pH meter (WTW, Berlin, Germany) was used. A TitroLine Easy potentiometer (SI Analytics GmbH, Mainz, Germany) was used for the determination of amino nitrogen. Bentonite clay was investigated for its mineral and chemical content using the X-ray diffraction technique and FTIR analysis. Table 2 presents the chemical and mineralogical characterization of native bentonite used in this study.

### 2.2. Preparation of Sorbents

For this synthesis, double-distilled water and 2 M sodium chloride solution (bought from Chim Reactiv Bucharest, Romania) were used. A total of 10 g of bentonite (BtB) was put in contact with a 2 M sodium chloride solution. This mixture was carried out under continuous stirring for 4 h at a temperature of 80 °C. The next step was washing by dialysis with bi-distilled water until the complete removal of chlorine ions [42]. Determination of chlorine concentration was determined using the sodium thiosulphate titration method using a redox electrode. Acetate buffer and potassium iodide are added to the sample, resulting in the formation of iodine upon reaction with chlorine. This process was performed in triplicate. At the third repetition, the bentonite was filtered by centrifugation. During filtration, the first layer (first fraction, Na-BF1), the one on the bentonite surface, was sampled. Separately, the next layer, the middle layer (second fraction, Na-BF2), and the bottom layer (third fraction, Na-BF3) were extracted. After the three layers of bentonite were taken separately, they were subjected to drying for 4 h at temperatures of 80–120 °C. For the preparation of the four bentonite sorbent solutions (BtB, Na-BtF1, Na-BtF2, and Na-BtF3) of 5% concentration, double-distilled water was used. These were homogenized in an orbital shaker-type GFL Shaker 3015 (bought from ProfiLab24 GmbH, Berlin, Germany) for 2 h at a temperature of 19 °C. These solutions were added to the wine.

The bentonite samples were introduced into the wine by moistening them and forming 5% concentration solutions. For the preparation of the four bentonite samples (BtB, Na-BtF1, Na-BtF2, and Na-BtF3), double-distilled water was used, then homogenized in an orbital shaker-type GFL Shaker 3015 (bought from ProfiLab24 GmbH, Berlin, Germany) for 2 h at a temperature of 19 °C.

### 2.3. Treatment of Sauvignon Blanc Wine Samples

Sauvignon Blanc is a dry, young white wine that presents a translucent greenish-yellow color and a fine balanced taste, with a complex bouquet of tastes, being appreciated as a type of wine of superior quality. Twenty-one wine samples of 10 mL each were prepared at 19–21 °C and filtered. For all four clay adsorbents, five wine samples were allocated, and the 21st wine sample was considered as the control sample. Five samples were treated with unmodified bentonite BtB, five with Na-BtF1, five with Na-BtF2, and the last five with Na-BtF3. Clay solutions prepared at a concentration of 5% each were added to the wine samples in different volumes: 0.1, 0.2, 0.3, 0.4 mL, and 0.5 mL sorbent/10 mL wine. After mixing, the samples were centrifuged at 350 rpm for 5 min, immersed for 48 h in the cold (1–3 °C), and then heated to the temperature of 19–21 °C.

**Figure 1:**
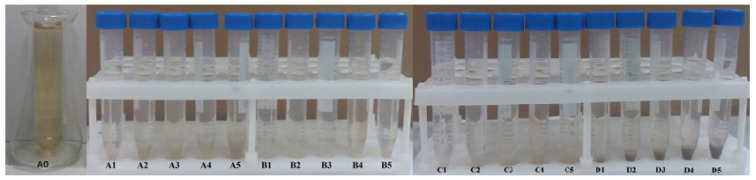
Visual/sensory analysis: (**A0**)—Sauvignon Blanc wine sample; (**A**)—BtB; (**B**)—Na-BtF1; (**C**)—Na-BtF2; (**D**)—Na-BtF3; numbers (1–5) in different volumes: 0.1, 0.2, 0.3, 0.4, and 0.5 mL sorbent/10 mL wine.

In Figure 1, some differences can be observed for the series of wine-treated bentonite fraction 1 (Na-BtF1) in the clarity of wine samples with concentrations of 0.1–0.3 mL sorbent/10 mL wine sample. At a concentration of more than 0.3 mL of sorbent, there is an increase in pH. In the case of the wine treated with Na-BtF2, stabilization and clarification of the wine structure are observed up to the concentration of 0.3 mL of sorbent/10 mL of wine sample, but starting from this volume of sorbent, the beginning of the oxidation phenomenon can be observed. For the series of Na-BtF3 samples, it is observed, even from the lowest sorbent concentrations, that the phenomenon of flocculation of the clay sample in the wine appears.

### 2.4. Protein Stability Test

To reduce the risk of organic deposits forming in white wine during the pre-bottling and storage stage, different working protocols have been started and developed with the aim of finding protein stability. Protocols known and presented in Table 1 can be combined depending on the goal pursued during the wine protein stability process (for example, thermal stability + tannin, time–temperature thermal stability + organo-inorganic stabilizing compounds).

The thermal stability test at various temperatures is one of the most common methods performed in winemaking laboratories. This test consists of heating the wine sample at a high temperature for a period of time in order to destabilize the protein compounds, thus resulting in protein aggregation [43]. This type of test is preferred because it can predict whether the wine is prone to protein aggregation, thus deciding the relative degree of protein instability. The working protocol for performing this type of test is not a standardized one [2,44]. According to Ledoux V. and Dubourdieu, D. [39,40], there are several variants of the same procedure with large time variations (5 min–18 h), separated or interspersed (for example, 80 °C for 30 min or heating the wine to 90 °C for 60 min + cooling the wine sample at 4 °C for 6–18 h), these being chosen and adapted according to the intended purpose.

In this work, the protein stability test at room temperature was used. After the cold immersion of the samples, the control sample and the 20 series of wine treated with the four clay-based adsorbents (BtB, Na-BtF1, Na-BtF2, and Na-BtF3) were heated to a temperature of 19–21 °C, for 25 min. The protein level in the treated white wine was analyzed using spectrophotometry.

### 2.5. Spectrophotometric Analysis for Protein and Polyphenolic Compounds

A UV spectrophotometer was used to find the content of protein and polyphenolic compounds in the twenty-one wine samples (including the control sample). Reading the absorbance at the wavelength of 280 nm (proposed by researcher Ribéreau-Gayon as the best predictor for the identification of polyphenols in wine). To estimate the Total Polyphenolic Index (IPT, Equation (1)), the ability of aromatic rings to absorb UV light will be taken into account (these rings belong to the majority of polyphenolic compounds) [45,46].

IPT = A280 nm · N(1)

where A280 nm—absorbance at the wavelength of 280 nm; N—degree of dilution of white/red/rosé wine.

In the case of total phenolic content (TFP), these are reported as Eq. Gallic acid, mg/L. To find the TFP, the wavelength of 280 nm is considered, with a correction of four units from the index, according to Equation (2). This correction is taken into account because, at the wavelength of 280 nm, other compounds are also present, such as nucleotides, proteins, and peptides [47,48]. Hydroxycinnamic substances (SFC, Equation (3)) and flavonoid ones (SFF, Equation (4)), reported as Eq. Caffeic Acid and Catechin, mg/dm^3^, respectively, can be determined using the absorbance at wavelengths of 280 nm and 320 nm with a correction index of 1.4 [48].

C_SFT_ = (N · A280 nm − 4) · 29.5 (2)

C_SFC_ = (N · A320 nm − 1.4) · 10 (3)

C_SFF_ = (N · A280 nm − 4) − 2/3 2/3(N · A320 nm − 1.4) (4)

where TFP—the content of total polyphenolic substances, expressed in Eq. Gallic acid, mg/L; SFC—the content of cinnamic polyphenolic substances, expressed in Eq. Caffeic Acid, mg/L; SFF—the content of flavonoid polyphenolic substances, expressed in Eq. Catechins, mg/L; A280 nm—absorbance at the wavelength of 280 nm; A320 nm—absorbance at the wavelength of 320 nm.

### 2.6. Determination of the Degree of Oxidizability

**Scheme 1:**
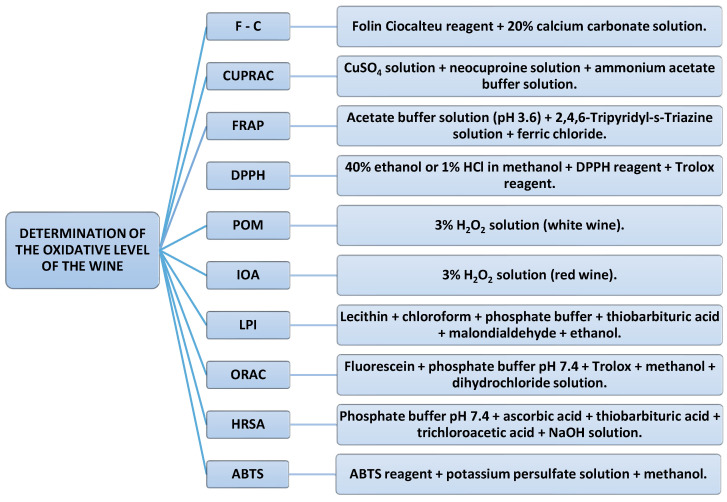
Most widely used known methods for finding the oxidizability of wines.

The content of polyphenolic acids, especially cinnamic acids, must be limited to the maximum because they can be easily oxidized to caftaric quinones. The presence of caftaric quinones in wine leads to the appearance of the browning defect of the wine (it changes its color to dark brown), and they can also react with the thiol compounds, thus changing the wine’s aroma. To remove this inconvenience from wines, different working protocols have been proposed to expect the oxidative degree of the wine. The most well-known work protocols (Scheme 1) for determining the oxidability of wine and analyzed spectrophotometrically are the following: hydroxyl radical-scavenging activity (HRSA), lipid peroxidation inhibition (LPI), Folin–Ciocalteu (F-C), maderization, the oxidative test of polyphenols (POM), the anthocyanin oxidizability index (IOA), the ferric antioxidant power reduction test (FRAP), the 2,2′-diphenyl-1-picrylhydrazyl (DPPH) radical test, the 2,2′-azinobis-(3-ethylbenzothiazoline-6-sulfonate) (ABTS), oxygen radical absorbance capacity (ORAC), and copper antioxidant capacity reduction test (CUPRAC) in wine [10,20,21,47].

In this work, the POM-type oxidation test was used. The working protocol for this test consists of introducing a 3% hydrogen peroxide solution (H_2_O_2_) into the 20 treated wine solutions, then heating them to a temperature of 60 °C for 60 min [16,33]. At the end, the wine samples were cooled to a temperature of 19–20 °C and subjected to spectrophotometric analysis. For seeing the degree of oxidation described by Equation (5), the wavelengths of A_420_ nm and A_520_ nm were considered [18,20].

(5) $${POM}_{test(\%)}=\frac{A_{420_{\left(wine+H_{2}O_{2}\right)}}-A_{420_{\left(wine\right)}}}{A_{420_{\left(wine\right)}}}\cdot 100$$

### 2.7. Determination of Assimilable Nitrogen Compounds in Wine

The estimation of the amount of assimilable nitrogen in the mini-samples of white wine was performed by the formalin/Sorënsen method, divided into two mini-stages. The first part of this protocol consists of the addition of 10 mL of double-distilled water over the wine sample and titration with 0.1 N NaOH solution in the wine to a pH value of 6.8. Add 8 mL of formalin mixture. The second part of the work protocol consists of continuing to titrate the wine solution with the same basic solution until the final solution in the glass reaches a pH of 9.1.

The determination of the volume of amino nitrogen (CN, mL/L wine) consists of the volume of NaOH solution (V_NaOH_, mL) added to the wine after the addition of the formalin mixture to the wine sample. For the CN calculation, it is considered that 1 mL of NaOH of 0.1 N concentration corresponds to 1.4 mg of amino nitrogen, along with the correction coefficient (k_NaOH_) according to Equation (6). The amino nitrogen content is expressed in mg/L in the analyzed wine sample.

(6) CN=VNaOH·1.4·kNaOH·1000Vpv

### 2.8. Characterization of the Prepared Sorbents

The four bentonite samples used in this work were characterized by Brauner–Emmet–Teller and Barrett–Joyner–Halenda (BET-BJH) methods performed at the Institute of Chemistry in Chisinau, Republic of Moldova. The Autosorb-1 MP device (Quantachrome, Boynton Beach, FL, USA) was used. FTIR-ATR analysis was made using an Agilent Technologies Cary 630 FTIR purchased from Santa Clara, CA, USA, in the spectral range of 4000–500 cm^−1^. The smectite materials were analyzed by the X-ray diffraction method (XRD), and the apparatus used was a Mini Flex 300–600 diffractometer purchased from Rigaku Corporate, Tokyo, Japan, (45 kV, 15 mA 2.0000 degmin, X-ray Cutubes).

## 3. Results

### 3.1. BET-BJH Analysis

**Figure 2:**
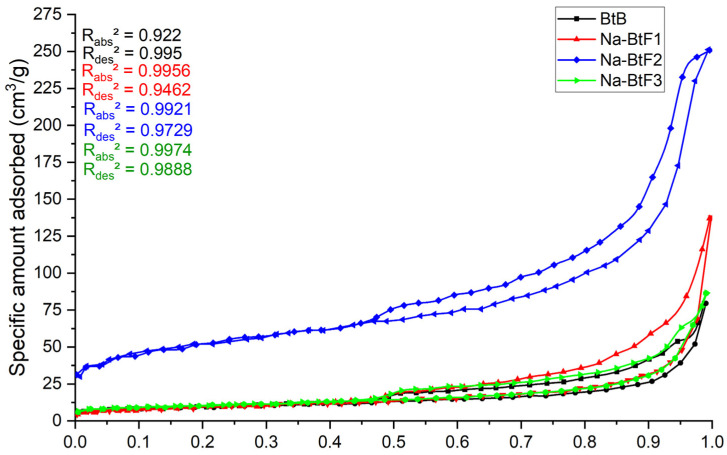
Adsorption–desorption isotherms of nitrogen.

In Figure 2, the adsorption–desorption isotherms are presented. According to IUPAC, they are of type IV, specific to mesoporous absorbent materials.

According to the IUPAC classification of hysteresis forms for BtB, Na-BtF1, and Na-BtF3, they correspond to the H3 type; this type of hysteresis is provided by the presence of interstitial pores due to aggregates of particles in the sheets. The modified Na-BtF2 material corresponds to H4-type hysteresis, a form attributed to capillary condensation in the interstitial pores due to particle aggregation. The determination coefficient R^2^ for all the absorption and desorption curves presented in Figure 2 is over 0.9, meaning that the results obtained have a very high correlation. Following BtB modification, the specific surface increased six times for Na-BtF2 (Table 3), and for Na-BtF3, the increase was from 34 m^2^g^−1^ to 37 m^2^g^−1^. In the case of the Na-BtF1 material, a decrease in the specific surface area of about 10% is observed.

**Figure 3:**
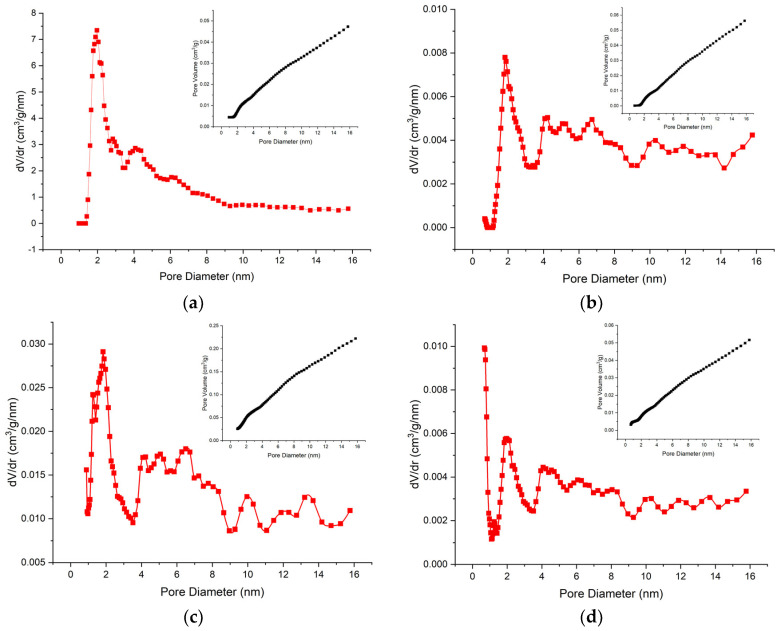
Characterization analysis by the BJH method for (**a**) BtB, (**b**) Na-BtF1, (**c**) Na-BtF2, and (**d**) Na-BtF3.

According to the data presented in Table 3, a decrease in the number of mesopores is seen for Na-BtF1 and Na-BtF3; a possible explanation can be condensation of adjacent layers of microporous silicon. In the case of Na-BtF2, an increase in meso- and micro-pores is seen. To complete this textural analysis for the bentonite samples, a series of determinations were made about the size of the pores and their distribution in the meso-porous region found by the BJH method. From these results, the first mesopores of BtB (Figure 3a), respectively, and the modified ones have sizes between 4 and 9 nm (Figure 3b–d).

The decrease in the characteristic peaks in the mesoporous region (2–15 nm) shows the decrease in the number of mesopores for Na-BtF1 (Figure 3b) and Na-BtF3 (Figure 3d); an explanation would be condensation of adjacent silica layers. In the case of Na-BtF2 (Figure 3c), a peak increase of 2 nm is seen; this means that there has been an increase in meso- and micro-pores for Na-BtF2.

### 3.2. XRD Analysis

**Figure 4:**
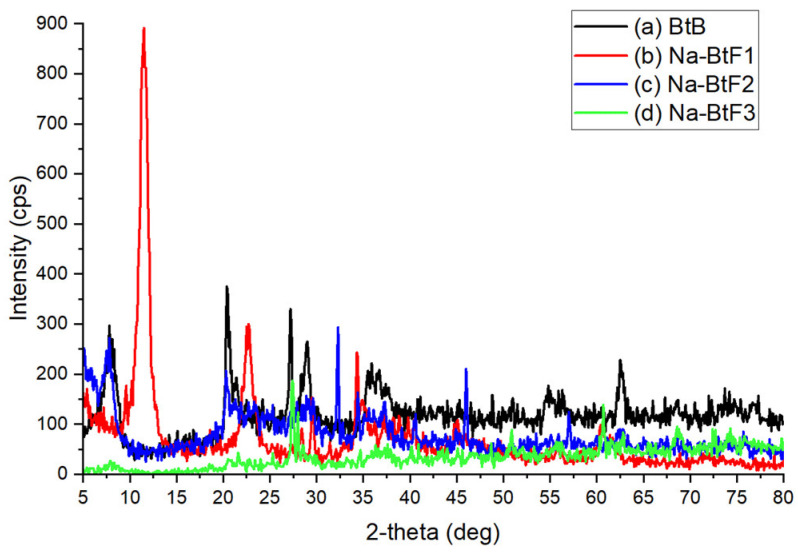
XRD analysis of (a) BtB, (b) Na-BtF1, (c) Na-BtF2, and (d) Na-BtF3.

The diffractogram corresponding to the autochthonous bentonite BtB, respectively, and the modified ones (Na-BtF1, Na-BtF2, and Na-BtF3) are presented in Figure 4. The components found in the bentonite samples were montmorillonite, cristobalite, quartz, albite, feldspars, calcite, dolomite, and pyrite.

These modifications of the bentonite samples using sodium ions (Figure 4) led to an increase in the basal d_001_ distance for Na-BtF1, leading to values greater than 2-theta. However, the sharp XRD reflection decreases for Na-BtF2 and Na-BtF3; an explanation for this can be the modification of their micro-pores. Peak intensity for montmorillonite (5 deg area) for Na-BtF1 after modification of BtB with sodium ions and peak intensity of quartz peaks decreased. Conversely, for Na-BtF3, the presence of quartz is high.

### 3.3. FTIR-ATR Analysis

**Figure 5:**
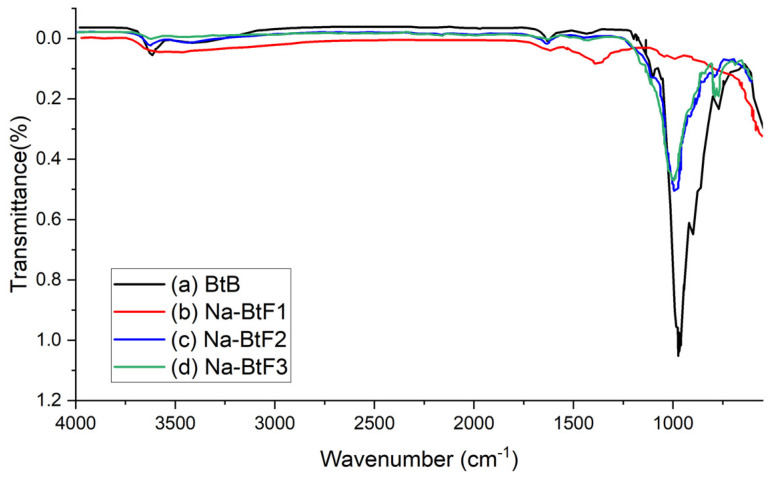
Results of FTIR-ATR analysis of (a) BtB, (b) Na-BtF1, (c) Na-BtF2, and (d) Na-BtF3.

To see the effect of cation exchange with sodium ions on the functional groups of BtB, FTIR-ATR spectra were performed in the range of 4000–500 cm^−1^ as a common approach to finding the characteristics of functional groups [49]. The vibrations, respectively, the bending modes for the hydroxyl groups and the Si-O stretches in the bentonite structure, are observed in the range of wavelengths 3745–3115 cm^−1^ and 1227–553 cm^−1^ [50]. The wavelength range of 3716–2800 cm^−1^, highlighted in Figure 5, corresponds to the removal of hydroxyl groups from the BtB structure [51]. The decrease in intensity around the area 3724–3523 cm^−1^ for Na-BtF1 shows the decrease in the interlayer absorption capacity; the wavelength shift in the range 3730–3600 cm^−1^ may show a dissolution of the octahedral layers performed during the cationic exchange of BtB. The same situation for Na-BtF1 is also clear in the case of wavelengths of 1200–750 cm^−1^ and 770–671 cm^−1^. These ranges correspond to Si-O-type vibrations. The Na-BtF1 sample shows two absorption maxima at 1646 cm^−1^ and 1386 cm^−1^, characteristic of the presence of interlayer H-O-H groups [52]. The specific Si-O stretching peaks of quartz and silica coincide at absorption maxima 770 cm^−1^, and the Si-O stretching vibration of silica occurs in the region of 673 cm^−1^. In the range of 3640–3610 cm^−1^, the modified samples (Na-BtF1, Na-BtF2, and Na-BtF3) present a different intensity peak compared to BtB. This range is associated with the OH group coordinated by the sodium cations. The vibrations present in BtB around 3420 cm^−1^, are OH groups involved in H_2_O-H_2_O bonds; Figure 5 shows how this area has decreased, until the curve flattens. The same aspect is visible in the range 3416–3127 cm^−1^, a region attributed to the H_2_O bending vibration.

The bending mode for H_2_O is visible in the range 1637–1625 cm^−1^, an intense peak being recorded around 1634 cm^−1^ for Na-BtF2, and a flattening of the zone is at Na-BtF1. An explanation is the difference in coordination within the structure and hydration shell for each modified sample. The most pronounced peaks are located in the area of 985–967 cm^−1^, probably due to the Si-O-Si stretching mode, the most intense being BtB, but in the spectra of the modified bentonite samples, a significant decrease is found for two of the three materials, namely, Na-BtF2 and Na-BtF3. In the case of the Na-BtF1 material, a flattening of the curve is observed.

In the peaks area of 617–630 cm^−1^ and 780–795 cm^−1^, specific to the presence of cristobalite [53], significant changes are seen for the modified samples by decreasing the intensity of the peaks. The most visible spectrum is for Na-BtF1, which means that the presence of cristobalite and crystalline silica is low. This intensity is diminished along with increasing hydrogen ion concentration due to the transformation of the tetrahedral structure of BtB. The same aspect is visible in the case of Na-BtF2. However, for Na-BtF3, triple peaks at 798 cm^−1^, 792 cm^−1^, and 781 cm^−1^ are seen in this area of cristobalite, which could suggest the increase in impurities in this sample relative to the low volume of Na ions, compared to BtB.

### 3.4. Physicochemical Parameters for Sauvignon Blanc White Wine

**Figure 6:**
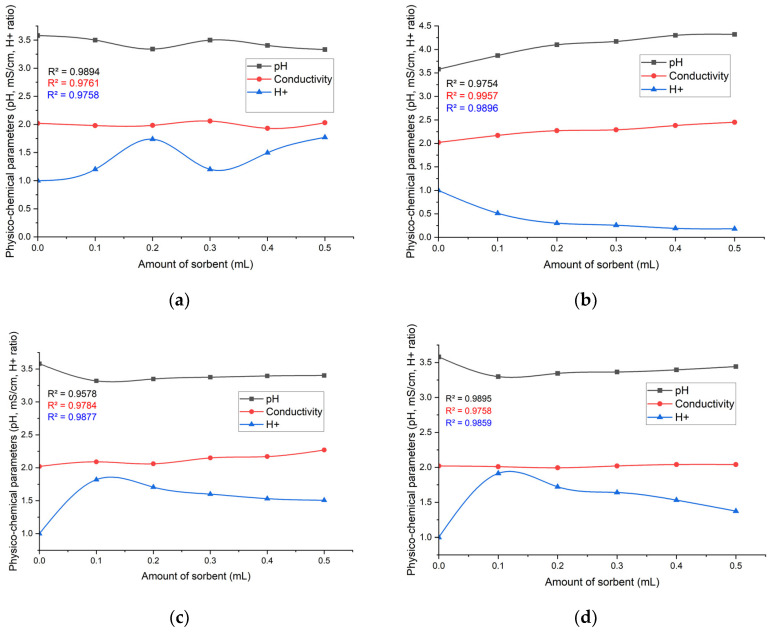
Results of pH, conductivity, and hydrogen ratio determinations for Sauvignon Blanc wine samples treated with (**a**) BtB, (**b**) Na-BtF1, (**c**) Na-BtF2, and (**d**) Na-BtF3.

Following the treatment of white wine with BtB, it is seen that the ratio of hydrogen ions increased, and the pH and conductivity decreased (Figure 6a). An explanation is the high content of organic acids in Sauvignon Blanc wine samples (tartaric acid). Among the four sorbents used, the wine treated with Na-BtF1 shown in Figure 6b stands out due to the low hydrogen ratio, and the pH and conductivity of the treated wine increased with the increase in the volume of bentonite. An explanation is the release of different elements from the bentonite (sodium ions).

**Figure 7:**
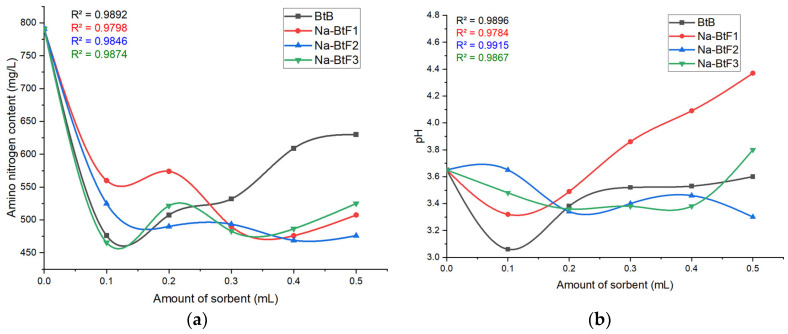
Amino nitrogen content (**a**) and pH value at the time of potentiometric determination (**b**) in Sauvignon Blanc white wine samples.

In the case of the wine treated with Na-BtF2 (Figure 6c) and Na-BtF3 (Figure 6d), it is observed how the pH decreased, and the ratio of hydrogen ions increased; the conductivity of the wine is constant in the dispersed medium, which means increasing the acidity of the white wine. This is further empowered by using the determination coefficient R^2^ with the experimental results. Next, the content of amino nitrogen (Naminic) was conducted potentiometrically, with a volume of 791 mg/L in the control sample (Figure 7a).

For the white wine samples treated with Na-BtF2 and Na-BtF1, a decrease of up to 40% of the initial volume of Naminic is observed at concentrations of 0.1–0.3 mL sorbent/10 mL (the concentration of 0.4 mL/10 mL wine being saturation point). Although the first two fractions of BtB are effective in wine on Naminic, analyzing the experimental results shown in Figure 6b and Figure 7b, it is observed that Na-BtF1 has an alkalizing effect (pH = 4.37) and Na-BtF2 helps to increase the intrinsic pH of wine (pH = 3.65) up to 3.3 (optimal pH). Applying the Pearson correlation coefficient formula on both the pH and amino nitrogen content presented in Figure 7, we can observe that the determination coefficient R^2^ is over 0.9, meaning that the results obtained have a very high correlation.

After treating the wine with Na-BtF3 (Figure 7a) at the concentration of 0.1 mL sorbent/10 mL wine, a lower content of Naminic of up to 42% is seen. According to Figure 7b, the pH of the wine samples treated with BtB, at concentrations of 0.2–0.4 mL sorbent/10 mL wine, is almost constant around the value of 3.5 units, while for the samples of wine treated with Na-BtF3, at concentrations of 0.2–0.3 mL sorbent/10 mL wine, the pH is 3.3 according to (Figure 7b).

**Figure 8:**
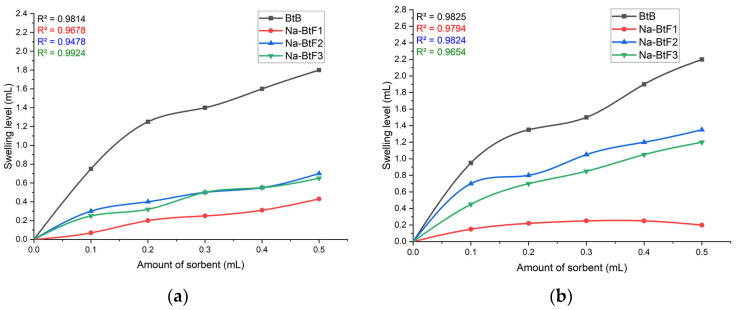
Sediment content present in Sauvignon Blanc white wine samples (**a**) after 20 min; (**b**) after 200 h.

According to the results shown in Figure 8a,b, the effect of Na-BtF1 on white wine is observed at concentrations of 0.2–0.4 mL/10 mL wine. The degree of swelling decreased, compared to BtB, in a percentage of approximately 83% (after 20 min, respectively, after 200 h of contact bentonite sample in wine). In the case of wine treated with Na-BtF2, at concentrations of 0.1–0.4 mL/10 mL of wine, compared to BtB, the swelling level decreased by 65% (after 20 min), and after 200 h 41%, at the concentration of 0.2 mL/10 mL wine. In the case of the wine sample put in contact with the Na-BtF3 absorbent, the swelling level decreased by 74% after one hour at the concentration of 0.2 mL/10 mL wine.

### 3.5. Effect of Amount of Adsorbent

**Figure 9:**
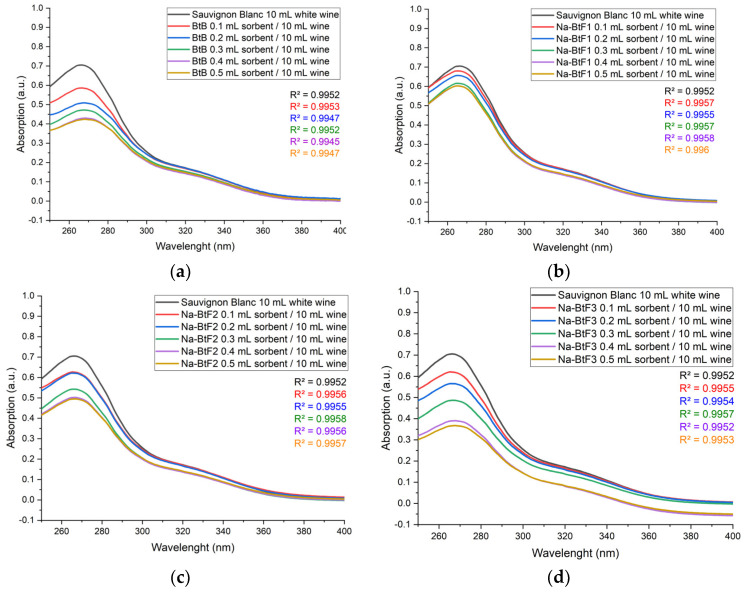
Results of spectrophotometric analysis in the UV range after treating white wine with (**a**) BtB, (**b**) Na-BtF1, (**c**) Na-BtF2, and (**d**) Na-BtF3.

According to (Figure 9), an adsorption maximum can be seen around 260 nm, the area dominated by phenolic substances. In the case of white wine, the main phenolic substances are hydroxycinnamic and their esters. These compounds are found in the 300–350 nm range. A complex, combined spectrum of the superposition and additivity of the individual absorption spectra, according to Firordt’s law, is often seen. The intensities of the 260–280 nm and 310–350 nm spectra can supply primary information about the content of C_6_-C_3_ (cinnamic) and C_6_-C_3_-C_6_ (flavonoids) groups, respectively, about the content of total polyphenols. The high intensity at 310–340 nm denotes an increased weight of hydroxycinnamic in the total polyphenolic complex. This aspect must be considered in the technological treatments of the wine because an exaggerated content of the cinnamic acids increases the risk of their oxidation accompanied by the browning of the wines. By treating Sauvignon Blanc wine with BtB, an adsorption maximum was found around 270 nm wavelength (Figure 9a). In this zone are the total polyphenolic substances and the proteins of the wine. In the wavelength range of 300–330 nm, it can be observed that the wine is less prone to oxidation after its treatment with 0.4–0.5 mL of sorbent/10 mL of wine. In the case of the wine treated with Na-BtF1 (Figure 9b), a uniform absorption of the proteins and total polyphenols of the wine (catechins and flavones) is seen at the concentrations of 0.1–0.3 mL sorbent/10 mL wine, 0.4 mL sorbent/10 mL wine being the saturation point. In the 320–370 nm area, a decrease in cinnamic phenolic substances (caffeic and ferulic acid) is seen, respectively. In the 350–400 nm area, the possible elimination of certain substances (polyphenols, anthocyanins) is seen. By treating white wine with Na-BtF2 (Figure 9c) at concentrations of 0.1–0.4 mL, uniform adsorption of the phenolic complex, compounds from the C_6_-C_3_ (cinnamic) and C_6_-C_3_-C_6_ (flavonoids) groups is seen; concentrations of 0.4–0.5 mL/10 mL represent saturation points. For the white wine treated with Na-BtF3 (Figure 9d), an increase in the adsorption of total phenolic compounds (SFT) and cinnamic compounds (SFC) is seen at 0.1–0.3 mL sorbent/10 mL wine; for 0.4–0.5 mL sorbent/10 mL wine, regression occurs in the region of polyphenolic compounds. Using the Pearson statistic on the absorption curves shown in Figure 9, it can be seen that the results obtained show a correlation coefficient of over 0.9.

**Figure 10:**
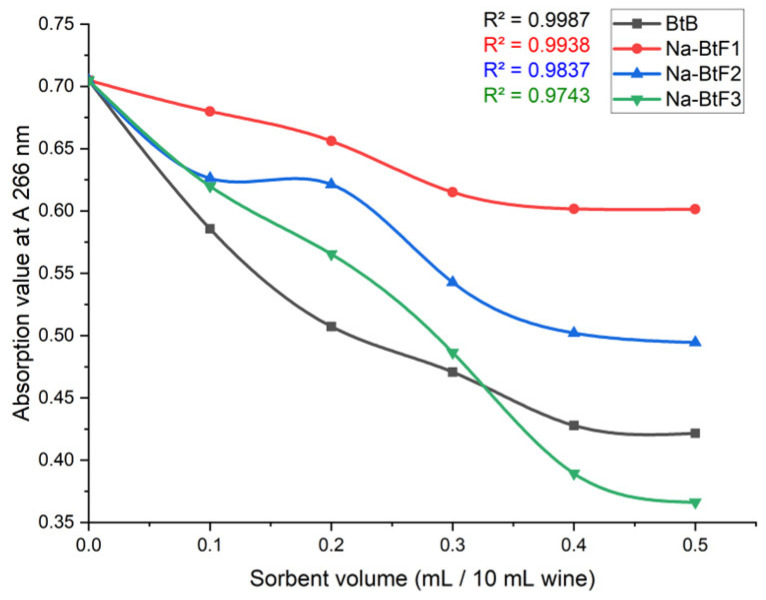
Maximum absorption at 266 nm absorbance for Sauvignon Blanc wine.

From the entire range of 250 to 270 nm, adsorption maxima are seen at the wavelength of 266 nm (Figure 10). The wine treated with Na-BtF1 and Na-BtF2 shows a uniformity of the adsorption of protein compounds followed by a stagnation of their adsorption. The saturation point is reached at concentrations of 0.4–0.5 mL sorbent. The best results were obtained in the case of using Na-BtF3, at c 0.4–0.5 mL sorbent/10 mL wine, with protein adsorption reaching 48%.

**Figure 11:**
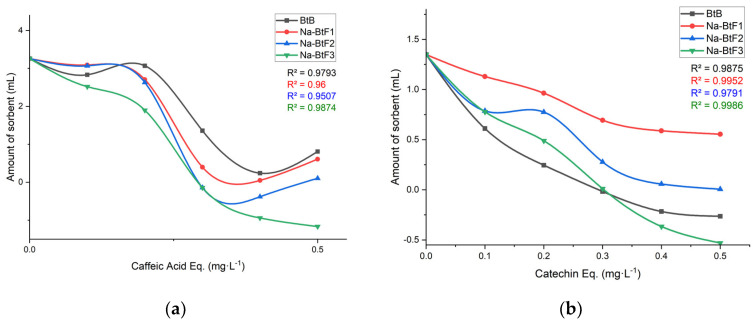
Impact of treatment of Sauvignon Blanc wine with bentonite on (**a**) SFC and (**b**) SFF.

The effect of Na-BtF3 on the composition of white wine at the concentrations of 0.1–0.2 mL sorbent/10 mL wine was that of decreasing the SFC content (Figure 11a), in percentage up to 41%, followed by a regression starting from the concentration of 0.3 mL sorbents; in the case of SFF (Figure 11b), the decrease was approximately 99%, at the concentration of 0.3 mL sorbent. After treating the white wine with Na-BtF1, with 0.4 mL of sorbent, the volume of SFC decreased (Figure 11a), in a percentage of 98.5%; SFF decreased in percentage of 58.9%, at the concentration of 0.5 mL sorbent/10 mL wine. For white wine treated with Na-BtF2, at concentrations of 0.4–0.5 mL, the SFF content decreased to 99.6%.

Next, the test was conducted to find the degree of oxidizability of the Sauvignon Blanc wine after treatment with the four series of bentonite. Therefore, to present the result of the POM-test more clearly, the analysis was performed for two absorbances, namely, the standard one of A420 nm and A520 nm, respectively (Table 4).

According to the POM-type oxidative test, the best result of the degree of oxidizability of the wine obtained at the wavelength of 420 nm was 88% in the case of its treatment with Na-BtF3, using a concentration of 0.2–0.4 mL sorbent/10 mL of wine. At the wavelength of 520 nm, the same order of magnitude (80%) was obtained using 0.3 mL sorbent/10 mL wine. Using the Na-BtF3 material at the concentration of 0.1 mL sorbent/10 mL wine, the best result was obtained in the case of reducing the amino nitrogen content.

## 4. Discussion

The pH, conductivity, and H+ shown in Figure 6a–d have the determination coefficient R^2^ over 0.9. This means that all the experimental results obtained have a very high correlation. This aspect is of interest because the wine must be kept at the best pH to inhibit pathogenic microorganisms such as Brettanomyces (these yeasts are difficult to remove from wine).

According to the protein stability test, the level of protein compounds in Sauvignon Blanc white wine was best recorded spectrophotometrically around the wavelength of 270 nm. The best retention efficiency was 48%, obtained by using 0.5 mL sorbent/10 mL wine from Na-BtF3-type modified bentonite.

According to the POM-test, the use of (a) BtB led to a reduction in the degree of oxidizability up to 85% 0.2–0.3 mL sorbent; (b,c) 90%, at the concentration of 0.4 mL sorbent, (d) Na-BtF3 showed the best results 91%, for the concentration of 0.3 sorbent/10 mL white wine. On total polyphenolic compounds (Table 4), the best results for SFT adsorption were for the wine treated with Na-BtF3 with 0.3 mL sorbent (after this concentration, SFT precipitation occurs), followed by Na-BtF2 with 0.3–0.5 mL sorbent, in a percentage of 99%. This information about the content of SFT in wine is of interest because these compounds have the role of helping colloidal stabilization (by coagulation of proteins) through their reducing character and preserving the specific organoleptic properties of white wine (color).

In parallel with the potentiometric determination, the intrinsic and treated wine pH was also measured. During the treatment with all sorbents, the value of the intrinsic pH 3.58 decreased by approximately 8%, the exception being the case when the Na-BtF1 sorbent was used at concentrations of 0.3–0.5 mL sorbent/10 mL wine when its value increased by up to 20%. The total polyphenol index decreased by more than 44% after treating Sauvignon Blanc wine with Na-BtF3 at the concentration of 0.5 mL/10 mL wine, followed by Na-BtF1 by 32% at 0.4–0.5 mL sorbent/10 mL wine. Using Na-BtF2 (of 0.4–0.5 mL/10 mL wine) and Na-BtF3, at the concentration of 0.3–05 mL/10 mL wine, the content of hydroxycinnamic substances decreased by over 80%. The content of flavonoid-type phenolic substances decreased by approximately 97% for Na-BtF2 in concentrations of 0.4–0.5 mL sorbent/10 mL wine and 99.2 Na-BtF3 at the concentration of 0.3 mL/10 mL wine.

In a dynamic system such as haze wine, in which there are substances with different molecular masses and states of aggregation, a series of phenomena occur at the interface between the particles and the solvent due to the varied energy state of the adjacent surfaces, resulting in a reserve of free energy, which causes an accentuated instability [54]. As a result, the systems thus formed are not thermodynamically stable with respect to the mass of the dispersion medium. The reduction in this free energy produces the stabilization of the colloids in the system, a process that can be achieved by decreasing the surface tension (σ) and/or the interphase area (S—the large surface between the hydrated bentonite lamellae). In the dispersed system of haze wine, the particle size of the dispersed phase is small, and the gravitational forces are negligible. The interactions between them are determined by attractive and repulsive forces acting at short distances (Van der Waals forces, electrostatic forces, steric forces, electrostatic forces). The stability of these dispersed systems is determined by the value of these interaction forces [55]. The determining factor of the colloidal instability in white wines is in special proteins, which, at the pH of the dispersion medium (the wine), are electropositive. Effective colloidal stabilization of haze wine is achieved by administering different oenological adjuvants like bentonite suspension, which reduces the content of unstable components such as proteins in the wine.

Bentonites have as their main substance montmorillonite, which is characterized by a lamellar structure and interstate spaces populated with different cations. Montmorillonite generally has a negative residual charge that can be compensated by cations in the interlayer space. The crystalline formations of montmorillonite in bentonite are not tightly bound together, and water can remove these formations, realizing the swelling phenomenon. Swelling increases the distance between parallel layers, thus greatly increasing the range in which physicochemical interactions involved in binding proteins (and other unwanted substances) to the clay mineral can take place [56,57]. Bentonite presents very strong surface phenomena (adsorption) in relation to its mass [58] generated by its colloidal character and negative electric charge. Administered in wine, bentonite disperses in colloidal form and has an overall electronegative character. The proteins in haze wine have a predominantly electropositive character and, in contact with the bentonite solution, form aggregates in the form of flakes, which will sediment. The mechanism of action consists of the flocculation of the proteins that are found in a dispersed state in the wine through the mutual neutralization of the bentonite-protein electrical charges [59]. Apart from the electrostatic forces of attraction between the system components, in the proximity of the bentonite layers, adsorption phenomena occur on the surface of the clay lamellae due to the formation of several types of physical–chemical bonds.

The intensity and speed of the flocculation process are dependent on the type of bentonite used, decreasing in the following order: activated bentonite > sodium bentonite > calcium bentonite. In terms of value, the adsorbed amount of proteins is within the limits of 30/85 mg or 46% of the total protein content per 1 g of bentonite. Sodium bentonite is generally preferred because it generates a more generous space between the lamellae after swelling and thus presents a better potential for adsorption and ion exchange phenomena. Bentonite that has undergone the swelling process behaves as a structure made of multiple lamellar formations, with a very large surface [56] negatively charged and capable of ion exchange with charges of the opposite direction from the wine. In the processes of “binding” protein molecules as well as other substances (responsible for the formation of haze phenomenon in wine) to the montmorillonite from bentonite, several interactions of a physical and chemical nature are involved, such as electrostatic interactions, ion exchange, van der Waals bonds, and so on [60]. Mainly, the positive sites of the proteins associate with the negatively charged portions of the swollen bentonite solution, thus forming complex clay–protein aggregates, which lead to the destabilization of the colloidal solution in the wine that creates the turbidity and to the realization of stable formations of the type of flakes. The flakes are then removed through the filtration processes, thus achieving clarification and stabilization of the wine.

## 5. Conclusions

The purification of native bentonite in the form of fractions led to its structural modification by removing impurities and increasing the level of montmorillonite, a statement confirmed by the results of the analyses BET-BJH, XRD, and FTIR-ATR.

The Na-BtF3 fraction led to obtaining the best results for the retention of excess protein and polyphenolic compounds responsible for mostly protein aggregation in wine, proving to be an absorbent material with suitable structural properties that can be used industrially in the treatment of white wine.

In conclusion, we consider that adsorbent—Na-BtF3 (which mainly contains montmorillonite) obtained from natural BtB clay has an important role in white wine clarification and stabilization. The Na-BtF3 material can be a suitable candidate for the retention of some compounds responsible for the appearance of the haze phenomenon in wine.

## Figures and Tables

**Table 1:** Materials used as stabilizing agents known up to now.

Test Type SP		Used Materials	Dosage in Wine	Purpose/Working Principle	References
Thermal stability	*Cold*	Sodium bentonite changed with Boltron dendrimers	10–50 mL sorbent/L white wine	- Induction of protein and polyphenolic complex precipitation in white wine; - Retention of polluting compounds in wine (e.g., phthalates);	[25,26,27,28,29]
	*Hot*	-	-	- Denaturation of wine proteins; - Induces precipitation of the wine; - Simulates the formation of protein turbidity;	[30,31]
Thermal stability (hot/cold) with inorganic organic materials		Egg albumin, blood albumin	50–250 mg/L red wine;	- Removal of unwanted substances (phenolic compounds) to prevent colloidal precipitation and reduce wine astringency;	[32,33,34,35]
		Milk casein	150–300 mg/L white wine, 7–10.5 mg/L	- For adjusting the wine’s flavor due to the ability of selective adsorption on tannin;	[36]
		Carrageenan	1.5–2% solution, 2 g in 200 mL wine	- Removing the proteins from the wine;	[7,37]
		Sodium bentonite (BS)	200–400 mg/L red + white wine	- Deproteinization of wine; - Clarification of white, red, rosé wines; - Stabilization against colloidal and - protein disturbances; - Ultrasound (US) effects on white wine protein stability;	[30,38]
		Calcium bentonite (BC)	700–2000 mg/L white/red wine		
		BS + BC	700–1500 mg/L white/red wine		
		Active coal	100–500 mg/L wine	- Retention of phenolic compounds in wine; - For the correction of oxidized white wines; - Removing some defects in the wine (taste, smell);	[38]
		Kaolin	5000–6000 mg/L wine	- Clarification of the wine and removal of excess proteins;	[38]
		Sodium alginate	40–80 mg/L wine	- Formation of electronegatively charged alginic acid colloidal particles; - They mutually flocculate with the proteins in the wine, electron positively charged; - Alginic acid flocculates and clears wines that are in excess of stabilization;	[38]
Trichloroacetic (TCA)		TCA solution	1 mL TCA solution concentration 55% in 10 mL wine	- Precipitation of the total proteins present in the wine;	[2,39,40]
With tannin	-	Oenological, ethereal alcoholic	5 mg/10 mL wine	- Protein precipitation during wine storage by binding to phenolic compounds; - Data on the amount of protein compounds in wine precipitated by tannins;	[5,26,40]
	Hot	Condensed tannin (TC)	0.5 mL tannin solution (TC/TH) in water with 10% ethanol		[30]
		Hydrolyzed tannin (TH)			
	Cold	Condensed tannin (TC)	5% TC/TH solution in water with 10% ethanol		
		Hydrolyzed tannin (TH)			
Bentotest		Phosphomolybdic acid (FA)	100 mL FA reagent in 10 mL wine	- Protein precipitation by neutralizing the protein charge; - Aggregation of proteins with the molybdenum ion;	[2,40]
Protochek (PC)		Reagent PC	It is added to wine in a ratio of 1:2	- The ability of proteins to interact quickly with a negatively charged polymer;	[30]
Prostab (PS)		Reagent PS	0.05–0.1 mL PS/L white wine	- Specific reaction to proteins that cause turbidity; - The intensity of the turbidity is directly proportional to the protein instability of the wine;	[30]
With inorganic solvents		Ethanol	3% solution in white wine	- Reduction in protein solubility; - Precipitation of less soluble protein fractions at the pH of the wine;	[2,26]
With mannoproteins		-	-	- Reducing the degree of turbidity of the wine; - Gives a concrete answer about the presence of proteins in wine after treatment with tannin and PC;	[30,39]
With inorganic salts		Ammonium sulfate	0.5–2 g/L white wine	- Precipitation of wine proteins.	[41]

**Table 2:** Chemical and mineralogical composition of BtB clay.

BtB %				
Chemical Composition		Mineralogical Composition		
Component	%	Component	Before Purification%	After Purification %
SiO_2_	67.98	Montmorillonite	67.98	99.00
Al_2_O_3_	14.5	Quartz	9.50	~0.50
MgO	2.15	Cristobalite	24.00	~0.50
Fe_2_O_3_	1.13	Illite	0.50	Traces
Na_2_O	2.15	Beidelite	-	-
K_2_O	0.60	Alkaline and alkaline terous mineral compounds	5.80	Traces
CaO	0.90	Amorphous substance	Traces	-
MnO	-			
PC * calcination losses	5.67			

* 0 < traces < 0.05.

**Table 3:** Identification of specific surface area for bentonite samples.

Bentonite Samples	Specific Surface Area (SBET, m^2^g^−1^)	Total Pore Volume (V_t_, cm^3^g^−1^)	Mesopore Volume (V_me_, cm^3^g^−1^)	Micropore Volume (V_mi_, cm^3^g^−1^)
BtB	34	0.131	0.307	0.003
Na-BtF1	32	0.213	0.213	0.0
Na-BtF2	189	0.392	0.362	0.030
Na-BtF3	37	0.134	0.130	0.004

**Table 4:** Total phenolic compound concentrations and oxidation behavior index (POM-test) at different absorbances (A420 nm and A520 nm).

Clays Samples	POM-Test 420 nm (%)					POM-Test 520 nm (%)				
	0.1 mL	0.2 mL	0.3 mL	0.4 mL	0.5 mL	0.1 mL	0.2 mL	0.3 mL	0.4 mL	0.5 mL
**Sauvignon Blanc**	91.07					62.35				
BtB	19.51	13.9	13.8	16.37	19.51	15.46	29.03	31.26	20.24	15.46
Na-BtF1	16.45	13.25	11.83	12.96	16.45	12.67	28.88	12.59	13.69	12.67
Na-BtF2	13.38	13.04	11.01	11.84	13.38	28.58	25.11	12.11	23.94	25.58
Na-BtF3	12.33	11.98	9.87	10.35	12.33	17.81	19.42	12.01	17.43	17.81
	IPT					SFT (Gallic Acid Eq. mgL^−1^)				
**Sauvignon Blanc**	5.57					46.20				
BtB	4.8	4.45	4.07	3.8	3.79	23.60	23.60	23.60	23.60	23.60
Na-BtF1	5.34	5.34	5.34	5.34	5.34	46.93	46.93	46.93	46.93	46.93
Na-BtF2	4.99	4.99	4.99	4.99	4.99	30.70	30.70	30.70	30.70	30.70
Na-BtF3	4.95	4.95	4.95	4.95	4.95	29.35	29.35	29.35	29.35	29.35

## Data Availability

All original data included in this article are available from the authors upon reasonable request. The data are not publicly available due to privacy.
